# Sepsis in mechanically ventilated patients with spinal cord injury: a retrospective analysis

**DOI:** 10.1038/s41393-018-0217-5

**Published:** 2018-11-09

**Authors:** Sebastian Weiterer, Sarah Frick, Christoph Lichtenstern, Andreas Hug, Florian Uhle, Markus Alexander Weigand, Guido Hundt, Benedikt Hermann Siegler

**Affiliations:** 1 Department of Anesthesiology, Heidelberg University Hospital, Im Neuenheimer Feld 110, 69120 Heidelberg, Germany; 2 Spinal Cord Injury Center, Heidelberg University Hospital, Schlierbacher Landstraße 200 a, 69118 Heidelberg, Germany

**Keywords:** Risk factors, Sepsis, Quality of life, Trauma, Epidemiology

## Abstract

**Study Design:**

Retrospective analysis.

**Objectives:**

Sepsis, one of the most frequent and life-threatening complications on intensive care units (ICUs), is associated with a need for mechanical ventilation (MV) as well as adverse respiratory outcomes in hospitalized individuals. However, it has poorly been investigated in patients with spinal cord injury (SCI); a population at high risk for pulmonary and infectious complications.

**Setting:**

Spinal Cord Injury Center, Heidelberg University Hospital.

**Methods:**

Over a 5-year period, 182 individuals with SCI requiring MV during their ICU stay were analyzed. Data assessment included demographics, medical characteristics, focus and causative pathogen of sepsis, length of stay, weaning outcomes, and mortality.

**Results:**

Sepsis was recorded in 28 patients (15%), containing a subgroup of individuals suffering from infectious SCI and co-occurring primary sepsis with *Staphylococcus aureus* as the predominant microorganism. In most individuals, sepsis was found as secondary complication, which was associated with pulmonary foci, Gram-negative bacteria, and high mortality. More than 80% of individuals with secondary sepsis required induction of MV due to respiratory failure. Furthermore, respiratory failure was found to be independent of sepsis focus, spectrum of causative pathogens, SCI etiology, or severity of injury. Subsequent weaning from the respirator was prolonged in more than 90% with a high proportion of weaning failure.

**Conclusions:**

Sepsis predominantly occurs as a secondary complication after SCI and is associated with detrimental outcomes. Although the lung is frequently affected as a failing organ, not all sepsis foci are pulmonary. Awareness of both actual sepsis focus and causative pathogen is central to initiate an adequate sepsis treatment.

## INTRODUCTION

Sepsis occurs in 30% of all patients in European intensive care units (ICUs), is one of the most frequent and life-threatening complications, and remains an interdisciplinary challenge [1]. The highly variable syndrome is characterized by a deregulated host response to infection, rapidly contributing to respiratory complications, organ failure, and death [2–4].

Sepsis can arise from virtually any type of infection and both incidence and clinical presentation vary between different patient populations [5]. Since infections are a common complication in individuals with spinal cord injury (SCI), a comparable risk to develop sepsis can be assumed in this population. With annual incidences ranging from 12.1 to 57.8 (traumatic injuries) and from 6.0 to 8.6 (non-traumatic injuries) cases per million European inhabitants [6–8], SCIs represent a relevant healthcare challenge, necessitating the potential need for life-long medical and social support [9]. While, on the one hand, the prognosis of spinal injuries is determined by the severity of neurological deficits, infections, on the other hand, seem to impair long-term neurological and functional outcomes [10–12] and have been reported as a main cause of death in individuals with SCI [13–15].

As the severest manifestation of an infection may lead to organ failure, sepsis necessitates urgent medical care including mechanical ventilation (MV). According to the recently updated International Guidelines for Management of Sepsis and Septic Shock published by the *Surviving Sepsis Campaign*, clinical management is based on rapid identification and, if possible, removal of the infectious focus (i.e., by surgery—“surgical source control”) as well as the timely initiation of antimicrobial therapies [16]. Since delays in anti-infective treatment have been associated with a significant increase in mortality [17, 18], administration of antimicrobials is recommended within the first hour after diagnosis of sepsis [16]. Unfortunately, frequent signs of infection might be absent after SCI, leading to a potential delay in diagnosis and treatment [19]. Therefore, awareness of the focus and most probable underlying pathogen in individuals with both SCI and sepsis is of particular interest for subsequent treatment initiation, including the administration of a calculated antimicrobial therapy.

In general, two scenarios are possible with regard to individuals with SCI: sepsis can either co-occur with SCI or can arise as a separate complication without direct connection to the initial incident. Since both settings might represent distinct medical emergencies, identification of clinical and microbial variations can support establishment of appropriate treatment strategies. Therefore, the goal of this study was to assess and compare characteristics as well as respiratory outcomes associated with sepsis that either (i) coincided with or (ii) occurred as a secondary complication after SCI. A key aspect of the analysis was to determine the distribution of foci and causative pathogens to improve sepsis management strategies in this particularly vulnerable patient population.

## METHODS

### Study design and data collection

Patients with SCI who were treated at the Spinal Cord Injury Center at Heidelberg University Hospital between 14 December 2009 and 12 December 2014 and admitted to the ICU requiring MV were included in data assessment. Data analysis was performed after approval of the ethics committee of the medical faculty of the Heidelberg University (Approval number S-019/2016) and in accordance with the principles expressed in the 1964 Helsinki Declaration and its later amendments. In addition to SCI-related parameters, evaluated data included gender and age, weight, and height to calculate body mass index as well as pre-existing comorbidities based on the Charlson comorbidity index (CCI) [20]. Furthermore, ICU-related cardiopulmonary complications, in-hospital-length of stay (LOS) and ICU-LOS, indication, and duration of MV as well as mortality were extracted from electronic or paper-based records. Neurological assessment at our site was performed as part of the clinical routine according to the International Standards for the Neurological Classification of Spinal Cord Injury (ISNCSCI), including determination of neurological level of injury (NLI). As a measure for SCI severity the American Spinal Injury Association Impairment Scale (AIS) was used [21, 22].

### Sepsis

International Classification of Diseases German Modification ICD-10-GM codes “R65.0!”, “R65.1!,” and “R57.2” were used to identify individuals with sepsis. Sepsis diagnosis was retrospectively confirmed based on medical records and according to international consensus guidelines [23], requiring the presence or suspicion of infection combined with a systemic inflammatory response. Furthermore, sepsis was termed “primary” if it coincided with an SCI within 48 h, or “secondary,” if it was diagnosed >48 h after onset of SCI as a complication. Sepsis focus and causative pathogens (defined as isolates from the infectious focus and underlying microbial cause of sepsis) were obtained from medical records as well as microbiological and radiographic data.

### Ventilator weaning classification

Ventilator weaning was classified according to the S2k-Guideline “Prolonged Weaning” criteria published by the German Respiratory Society [24] in reference to International Consensus Conference Statements [25]. “Simple weaning” (weaning category 1) was defined as unproblematic proceeding from weaning initiation to extubation with the first spontaneous breathing trial (SBT), while successful weaning and extubation within three SBTs or 7 days of MV after first failure was classified as “difficult” (weaning category 2). Weaning category 3 (“prolonged weaning”) comprised three subgroups: category 3a and 3b were defined as successful liberation from ventilator after at least three SBTs or more than 7 days of MV after first SBT failure without (category 3a) or with use of non-invasive ventilation (category 3b). Category 3c (weaning failure) was defined as hospital discharge with invasive MV (tracheostomy) or death.

### Statistics

Group comparisons were performed using Fisher’s exact test for categorical data and Mann–Whitney *U* test for continuous data. *P* values <0.05 were considered significant. Statistical analysis was performed using GraphPad Prism (Version 6.0 f, GraphPad Software, La Jolla, USA).

## RESULTS

### Patients’ characteristics

**Fig. 1:**
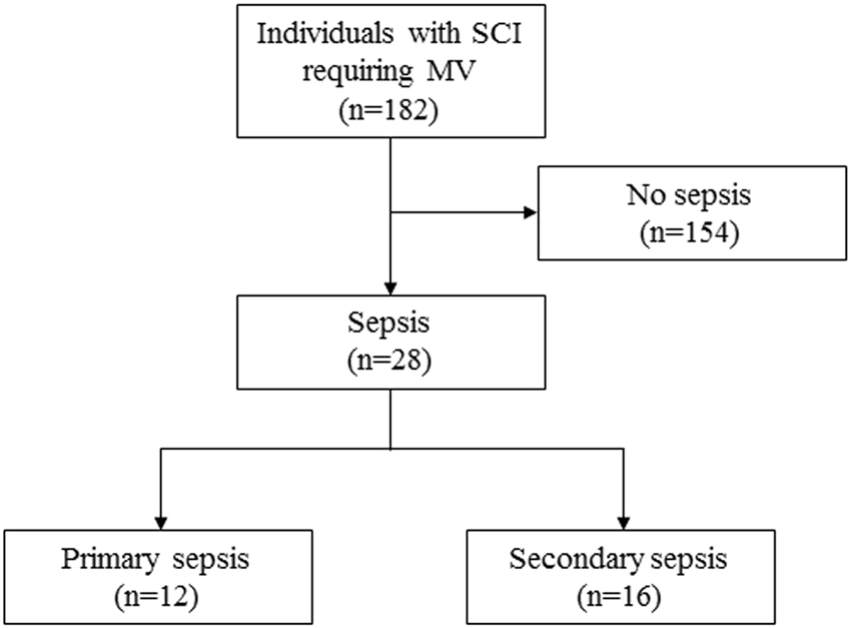
Study design. MV mechanical ventilation, SCI spinal cord injury

In total, 182 individuals with SCI requiring MV were included in the data assessment. Sepsis was recorded in 28 patients (15%) with a male-to-female ratio of 4.6:1 and a median age of 69 years (Fig. 1). In 12 of 28 individuals, sepsis was categorized as primary, coinciding with an infectious SCI. In contrast, secondary septic events occurred in 16 of 28 individuals with either traumatic or infectious SCIs. Time from SCI to diagnosis of secondary sepsis ranged from 4 to 8030 days (Supplemental Figure 3). Demographics and clinical characteristics of both primary and secondary sepsis cohorts are listed in Table 1. Mean (SD) CCI was 4 (2) in individuals with primary sepsis and 4 (3) in those with secondary sepsis. MV was initiated within 24 h after diagnosis of sepsis in 27 of all 28 cases. One patient was ventilated via tracheostomy prior to recognition of sepsis. The occurrence of secondary sepsis was significantly associated with respiratory failure as an indication for MV (81 vs. 0%; odds ratio (OR) 0.01, 95% confidence interval (CI): 0.00–0.22, *p* < 0.0001).

**Table 1:** Socio-demographic and clinical characteristics

Variables	Primary sepsis	Secondary sepsis	Test	OR	95% CI	*p* value
Gender (*n*)						
Male	10	13	Fisher’s exact	1.2	0.16–8.3	1.0
Female	2	3	Fisher’s exact	0.87	0.12–6.2	1.0
*Age (years)*						
Range	37–83	28–81	–	–	–	–
Mean (SD)	64 (14)	64 (15)	–	–	–	–
Median	69	67	Mann–Whitney *U*	–	–	0.86
*BMI (kg/m* ^*2*^ *)*						
Range	22–40	21–51	–	–	–	–
Mean (SD)	31 (6.8)	28 (7.8)	–	–	–	–
Median	32	27	Mann–Whitney *U*	–	–	0.27
*SCI etiology (n)*						
Trauma	0	10	Fisher’s exact	0.02	0.00–0.49	<0.001
Infection	12	6	Fisher’s exact	40	2.0–805	<0.001
*NLI (n)*						
C1–4	5	3	Fisher’s exact	3.1	0.56–17	0.23
C5–8	2	8	Fisher’s exact	0.20	0.03–1.2	0.11
T1–S5	5	5	Fisher’s exact	1.6	0.33–7.5	0.70
*AIS (n)*						
AIS A	1	6	Fisher’s exact	0.15	0.02–1.5	0.18
AIS B	2	7	Fisher’s exact	0.26	0.04–1.6	0.22
AIS C	6	2	Fisher’s exact	7.0	1.1–45	<0.05
AIS D	3	1	Fisher’s exact	5.0	0.45–56	0.29
*Comorbid conditions a) (n)*						
Myocardial infarction	0	1	Fisher’s exact	0.41	0.02–11	1.0
Congestive heart failure	2	2	Fisher’s exact	1.4	0.17–12	1.0
Peripheral vascular disease	1	1	Fisher’s exact	1.4	0.08–24	1.0
Cerebrovascular disease	0	1	Fisher’s exact	0.41	0.02–11	1.0
Chronic pulmonary disease	3	2	Fisher’s exact	2.3	0.32–17	0.62
Connective tissue disease	0	1	Fisher’s exact	0.41	0.02–11	1.0
Ulcer disease	2	1	Fisher’s exact	3.0	0.24 to 38	0.56
Mild liver disease	3	0	Fisher’s exact	12	0.56–262	0.07
Diabetes	4	5	Fisher’s exact	1.1	0.22–5.4	1.0
Moderate or severe renal disease	3	2	Fisher’s exact	2.3	0.32–17	0.62
Diabetes with end-organ damage	2	2	Fisher’s exact	1.4	0.17–12	1.0
Any tumor	2	4	Fisher’s exact	0.6	0.09–4.0	0.67
*Complications on ICU (n)*						
Myocardial infarction	0	4	Fisher’s exact	0.11	0.01–2.3	0.11
Cardiopulmonary resuscitation	1	4	Fisher’s exact	0.27	0.03–2.8	0.36
*Indication for MV (n)*						
Surgery for septic source control	12	2	Fisher’s exact	145	6.3–3317	<0.0001
Respiratory failure	0	13	Fisher’s exact	0.01	0.00–0.22	<0.0001
Severe thoracic trauma	0	0	Fisher’s exact	–	–	–
Neurological status	0	0	Fisher’s exact	–	–	–
MV prior to sepsis	0	1	Fisher’s exact	0.41	0.02 to 11	1.0
*Airway status prior to MV (n)*						
Natural airway	12	15	Fisher’s exact	2.4	0.09 to 65	1.0
Tracheostomy	0	1	Fisher’s exact	0.41	0.02 to 11	1.0

*AIS* American Spinal Injury Association Impairment Scale, *BMI* body mass index, *CI* confidence interval, *COPD* chronic obstructive pulmonary disease, *ICU* intensive care unit, *MV* mechanical ventilation, *OR* odds ratio, *OSAS* obstructive sleep apnea syndrome, *SCI* spinal cord injury, *SD* standard deviation

^a^Only comorbid conditions with at least one observation are reported

### Focus and causative pathogen

The majority of identified sepsis foci were spinal (14 of 28), followed by the lung (10 of 28). Causative pathogens were Gram-positive bacteria in 16 of 28 and Gram-negative bacteria in 11 of 28 cases. *Staphylococcus aureus* was solely detected as a causative pathogen in individuals with a spinal focus (OR 143, 95% CI: 6.3–3267, *p* < 0.00001), whereas *Pseudomonas* * aeruginosa* exclusively occurred in those with a pulmonary focus (OR 37, 95% CI: 1.8–780, *p* < 0.01).

Table 2 summarizes the distribution of foci and causative pathogens in the primary and secondary sepsis cohorts. Secondary sepsis was associated with pulmonary foci (OR 0.02, 95% CI: 0.00–0.49, p < 0.001) and Gram-negative bacteria (OR 0.01, 95% CI: 0.00–0.30, *p* < 0.0001), predominated by *Pseudomonas aeruginosa* and *Klebsiella* species. Supplemental Figure 3 illustrates the distribution of sepsis foci with regard to time from SCI to sepsis diagnosis in secondary sepsis.

**Table 2:** Focus and pathogen

Variables	Primary sepsis	Secondary sepsis	Test	OR	95% CI	*p* value
*Sepsis focus (n)*						
Spinal	12	2	Fisher’s exact	145	6.3–3317	<0.0001
Pulmonary	0	10	Fisher’s exact	0.02	0.00–0.49	<0.001
Abdominal	0	2	Fisher’s exact	0.23	0.01–5.3	0.49
Urogenital	0	1	Fisher’s exact	0.41	0.02–11	1.0
Soft tissue	0	1	Fisher’s exact	0.41	0.02–11	1.0
*Gram-positive bacteria (n)*						
Total	12	4	Fisher’s exact	69	3.4–1431	<0.0001
*Staphylococcus aureus*	10	1	Fisher’s exact	75	6.0–942	<0.0001
*Enterococcus* species	1	3	Fisher’s exact	0.39	0.04–4.4	0.61
*Streptococcus* species	1	0	Fisher’s exact	4.3	0.16–115	0.43
*Gram-negative bacteria (n)*						
Total	0	11	Fisher’s exact	0.02	0.00–0.39	<0.001
*Pseudomonas aeruginosa*	0	5	Fisher’s exact	0.08	0.00–1.7	0.05
*Acinetobacter* species	0	1	Fisher’s exact	0.41	0.02–11	1.0
*Escherichia coli*	0	2	Fisher’s exact	0.23	0.01–5.3	0.49
*Klebsiella* species	0	3	Fisher’s exact	0.15	0.00–3.3	0.24
*Fungi (n)*						
*Candida albicans*	0	1	Fisher’s exact	0.41	0.02–11	1.0

*CI* confidence interval, *OR* odds ratio

### Duration of ventilation, weaning, LOS and mortality

Duration of ventilation and weaning outcomes are listed in Table 3. Secondary sepsis was characterized by a mean (SD) duration of MV of 37 (27) days. In these individuals, no simple weaning was recorded, whereas weaning was categorized as prolonged in 15 of 16 cases (94%). In addition, weaning failure (category 3c) occurred in 7 of 16 individuals with a secondary septic event.

**Table 3:** Characteristics of mechanical ventilation

Variables	Primary sepsis	Secondary sepsis	Test	OR	95% CI	*p* value
*Duration of MV (days)*						
Range	0.5–55	2–96	–	–	–	–
Mean (SD)	16 (19)	37 (27)	–	–	–	–
Median	5.8	34	Mann–Whitney *U*	–	–	<0.05
*Weaning category (n)*						
Category 1 (simple)	6	0	Fisher’s exact	33	1.6 to 674	<0.01
Category 2 (difficult)	1	1	Fisher’s exact	1.4	0.08 to 24	1.0
Category 3 (prolonged)	5	15	Fisher’s exact	0.05	0.00 to 0.49	<0.01
Sub-category 3a	4	7	Fisher’s exact	0.64	0.14 to 3.0	0.70
Sub-category 3b	0	0	Fisher’s exact	–	–	–
Sub-category 3c	1	8	Fisher’s exact	0.09	0.01 to 0.88	<0.05

*CI* confidence interval, *OR* odds ratio

With regard to the indication of MV in secondary sepsis, there was neither a significant association with weaning classification nor with SCI etiology, severity of injury, sepsis focus, or causative pathogen. Supplemental Figures 1 and  2 provide further information about individuals with primary or secondary sepsis based on these parameters.

Neither mean (SD) in-hospital-LOS nor ICU-LOS significantly differed between both cohorts (primary sepsis: 131 (55) and 25 (20) days; secondary sepsis: 126 (89) and 49 (43) days). Five patients with sepsis, who were exclusively recorded in the secondary sepsis cohort, died in the ICU.

## DISCUSSION

Despite many efforts, sepsis remains one of the leading causes of death worldwide [26]. Those who survive are confronted with devastating long-term effects on morbidity and quality of life [27]. Since any delay in the initial treatment worsens overall prognosis in sepsis, awareness of both the most probable site of infection and the underlying pathogen is fundamental to ensure early and appropriate treatment [16]. However, while clinical appearances and outcomes of sepsis have been widely investigated in the general adult population, studies focusing on the rare subgroup of patients with coincidental SCI and sepsis are missing. To close this gap, we retrospectively assessed and compared clinical and microbial characteristics associated with sepsis in individuals with SCI. This study focused on patients with sepsis necessitating MV and comprised either sepsis presenting with onset of SCI or as a secondary, delayed complication. Our analysis revealed distinct distributions of septic foci and underlying pathogens as well as adverse clinically relevant outcomes.

Our study contained a group of individuals with infectious SCI and primary sepsis with *Staphylococcus aureus* as the predominant underlying microorganism, which is in line with reports showing pyogenic spondylodiscitis mainly associated with *Staphylococcus aureus* [28, 29]. Individuals with primary sepsis were exclusively de novo ventilated for surgical source control and underwent successful weaning in nearly all cases.

However, the majority of septic events in our study were found as delayed complications after SCIs of various etiologies. Since, in general, infectious complications are a common menace in the SCI population [30], but might also be occult due to neurological injury, the secondary development of sepsis as the severest manifestation of an infection is of particular interest. In line with sepsis in non-spinal cord-injured individuals [1, 31], the lung was the predominant focus in the secondary sepsis cohort. Among those patients with a pulmonary focus, only one individual was diagnosed with ventilator-associated pneumonia. Vice versa, more than one-third of secondary septic events developed from non-pulmonary infections. These included abdomen, spine, urinary tract, and soft tissue, which, in contrast to sepsis in general [1, 31], were evenly distributed among individuals with a non-pulmonary focus. This is an important aspect considering the optimal management of this unique population.

Remarkably, the majority of patients with secondary sepsis necessitated de novo and prompt induction of MV due to respiratory failure. The need for MV based on respiratory failure seems to be independent from sepsis focus, spectrum of causative pathogens, SCI etiology, or severity of injury. Our findings demonstrate that individuals with SCI do not necessarily suffer from a pulmonary sepsis focus when they show clinical signs of lung injury. In addition, respiratory failure could be misinterpreted as a pulmonary sepsis focus, because clinical signs leading to a sepsis focus outside the lung might be occult due to SCI-related impairments [32–34]. Of note, although urinary tract infections are among the most frequent conditions seen after spinal injuries [30], urosepsis played a minor role in our cohort of septic individuals requiring MV.

Complementary to identification of the actual sepsis focus, awareness of the most probable microorganism is necessary to effectively tailor any anti-infective management [5, 16]. Remarkably, with reported rates ranging from 22 to 37.4% [19, 35], individuals with SCI are at high risk to receive inadequate empiric treatments, which is associated with increased morbidity and mortality in the general sepsis population [17, 18, 36, 37]. Since coverage of likely pathogens critically impacts the effectiveness of treatment, we analyzed the spectrum of causative microorganisms in our study cohort. In significant contrast to primary events, Gram-negative bacteria were isolated in the majority of patients with secondary sepsis. This is in line with the predominance of Gram-negative microorganisms in the overall population of sepsis, as shown in a previous multicenter trial [31]. In addition, the identified spectrum of pathogens was similar to previous reports outside the SCI population, with *Pseudomonas aeruginosa*, *Klebsiella* spp., and *Escherichia coli* as the three dominant Gram-negative species [38].

As important consequences of secondary sepsis in patients with SCI, our analysis revealed adverse respiratory outcomes including long duration of MV and poor ventilator weaning. Several studies addressed the aspect of liberation from respirator in the general population of patients with sepsis [39, 40], pointing towards prolonged ventilator dependency as well as impeded weaning [40]. However, so far, valuable data on respiratory outcomes in individuals suffering from both sepsis and SCI are missing. Importantly, we found that weaning from the ventilator after the acute phase of sepsis was prolonged in more than 90% of individuals with a secondary septic event, with a high proportion of weaning failure. Moreover, a fatal outcome was found in almost one-third of patients with a secondary septic event. This is even higher than reported in a systemic meta-analysis of sepsis in general that found mortality rates ranging from 17 to 26% in the broad population of critically ill patients [26].

In summary, sepsis in individuals with SCI rather presented as a secondary complication than as a primary event. Secondary sepsis was associated with distinct clinical and microbial characteristics as well as a high mortality. The majority of these individuals needed induction of MV due to failure of the respiratory system. As an important finding, respiratory failure occurred independent from SCI etiology, severity of injury, sepsis focus, or causative pathogen. Since a typical clinical picture indicating a non-pulmonary sepsis focus might commonly be missing after SCI, our results point to a high risk of misinterpreting the failing lung as the infectious focus of sepsis in the population of people with SCI. This is of certain relevance, since any inadequate treatment, that is, by initial administration of antimicrobials that do not reach the actual focus—might worsen the prognosis of these critically ill patients.

Due to its character as a retrospective analysis at a single institution, we acknowledge several limitations. Since data were obtained from both electronic and paper-based medical records, automated extraction was not feasible. While ICD codes were used to identify patients with sepsis, no further information about disease severity (i.e., based on clinical scores) was available for retrospective analysis. The rarity of SCI in addition to the low rate of sepsis among the general hospitalized population explains the putative small number of sepsis cases in this study. Nevertheless, our data represent an important piece in the still incomplete picture of sepsis in individuals with SCI, underscoring the high risk of potential misinterpretation of clinical signs leading to delayed and/or inadequate treatments. Prospective multicenter trials are needed to further investigate predictors of sepsis after spinal injury, which might support the initial clinical management in this particular patient population.

## CONCLUSIONS

Our data are of particular interest for the clinical management of sepsis including source control and antimicrobial therapy, showing a heterogeneous clinical picture concerning site of infection and causative pathogen. Although respiratory failure is the most common indication for MV, consideration of non-pulmonary sepsis foci is central to initiate an adequate sepsis treatment.

## Electronic supplementary material

Supplemental Figure 3

Supplemental Figure 1

Supplemental Figure Legend

Supplemental Figure 2
