# Supplementary material for: Sepsis in mechanically ventilated patients with spinal cord injury: a retrospective analysis
Source: Spinal Cord. 2018 Nov 9;57(4):293–300. doi: 10.1038/s41393-018-0217-5 (PMC6477786; doi:10.1038/s41393-018-0217-5)
Supplement: Supplementary file 3 — Supplemental Figure Legend [file 41393_2018_217_MOESM3_ESM.docx]

**Supplemental Figure 1:** Indication for MV with regard to SCI-, sepsis- and weaning characteristics in primary sepsis. Abbreviations: AIS, American Spinal Injury Association Impairment Scale; Etiol, etiology; MV, mechanical ventilation; NLI, neurological level of injury; OR, odds ratio; OSAS, obstructive sleep apnea syndrome; SCI, spinal cord injury; SD, standard deviation. Each line represents one patient.

**Supplemental Figure 2:** Indication for MV with regard to SCI-, sepsis- and weaning characteristics in secondary sepsis. Abbreviations: AIS, American Spinal Injury Association Impairment Scale; Etiol, etiology; MV, mechanical ventilation; NLI, neurological level of injury; OR, odds ratio; OSAS, obstructive sleep apnea syndrome; SCI, spinal cord injury; SD, standard deviation. Each line represents one patient.

**Supplemental Figure 3:** Distribution of sepsis foci with regard to time from SCI to sepsis diagnosis in secondary sepsis. Abbreviations: SCI, spinal cord injury.
